# Maintaining Efficacy But Decreasing Toxicity in the Treatment of Hodgkin Lymphoma

**DOI:** 10.1097/HS9.0000000000000007

**Published:** 2017-12-20

**Authors:** Stephen M. Ansell

**Affiliations:** Division of Hematology, Mayo Clinic

Fifty years ago patients diagnosed with Hodgkin lymphoma (HL) had a dismal prognosis, but their outcome substantially changed with the introduction of combined modality therapy and the use of multiagent chemotherapy over subsequent decades.^1^ The initial use of treatment combinations such as ABVD (doxorubicin, bleomycin, vinblastine, dacarbazine) resulted in approximately two-thirds of patients remaining in remission 5 years posttreatment.^2^ Subsequent studies, however, have explored whether intensification of therapy results in a higher percentage of patients achieving a remission with initial treatment and whether the long-term prognosis of these patients is improved by a more aggressive treatment approach. Randomized controlled trials by the German Hodgkin Study Group (GHSG) have established escalated BEACOPP (bleomycin, etoposide, doxorubicin, cyclophosphamide, vincristine, procarbazine, prednisone) chemotherapy as a standard front-line treatment for advanced stage patients and have shown this combination to be highly effective.^3,4^

Initial studies established 8 cycles of escalated BEACOPP as the standard of care for advanced stage HL patients as shown in the GHSG HD9 trial.^4^ Concerns, however, existed that the intensive therapy resulted in more toxicity and long-term complications, and further studies therefore explored whether using fewer cycles of this combination could result in similar outcomes. The HD15 trial was subsequently performed comparing 3 arms: escalated BEACOPP for 8 cycles versus escalated BEACOPP for 6 cycles versus 8 cycles of baseline BEACOPP administered on a 14-day schedule.^5^ The initial report of the trial showed equally excellent results with fewer cycles of treatment and established 6 cycles of escalated BEACOPP as the standard of care. Longer-term follow-up, however, was needed to determine whether late toxicities and long-term adverse effects would in any way compromise the results seen in the original report.

In this episode of *HemaSphere*, Engert et al,^6^ provide long-term follow-up of the HD15 trial with follow-up now at 102 months. They report that the clinical efficacy seen in this study has been maintained. The 10-year progression-free survival is essentially the same between the 3 arms and is in the range of 81% to 84%. Furthermore, the 10-year overall survival is excellent and ranges between 88% and 92%. It is worth noting that these results are outstanding and provide an excellent outcome for patients with HL treated with initial therapy. A significant concern of an intensive treatment approach was that patients progressing on this intensive approach would not be able to be salvaged with subsequent treatments due to the fact that relapsing patients would be relatively chemoresistant. Based on the long-term results of this study, this does not appear to be true. In this trial, more than half of the patients appear to have been salvaged with further treatment, as the 5-year second progression-free survival is 54%.

One of the concerns with an aggressive approach has been the potential for long-term toxicities, and because of this there has been reticence by some groups to adopt a more aggressive chemotherapy approach. The long-term data from this trial does show that second malignancies are seen with a 10-year cumulative incidence of between 7% and 10%. Clearly, however, decreasing the number of cycles of treatment, and thereby abbreviating the intensity of therapy, does impact long-term complications. Giving 6 cycles of escalated BEACOPP, rather than 8, appears to almost half the standardized incidence ratio of second malignancies from 4.3% to 2.5%. The study's long-term follow-up is also important as the trial tested whether less radiation therapy would result in less toxicity. Radiation was only given to residual masses that were PET positive and this may also have had an impact on some of the long-term complications. Overall, however, this report convincingly confirms that 6 cycles of escalated BEACOPP maintains the intensity and efficacy of therapy and also decreases the frequency of long-term complications.

These follow-up data from the HD15 trial are really important for clinicians to understand the durability of benefit and also the potential long-term adverse effects of treatment. The results highlight the fact that intensive therapies are highly effective, but also confirm that intensive therapies result in some long-term complications. As one looks to the future, there will be opportunities to utilize novel agents, such as brentuximab vedotin or anti-PD1 antibodies, in the place of standard chemotherapy and hopefully maintain the significant benefits seen in trials such as the HD15 study, but minimize long-term complications. As patients live longer and are cured of their disease, it is important for us to continue to strive toward highly effective combinations with durable remissions, but with less toxicity.
